# Artificial Intelligence and the Clinical Gaze: Visual Practices of AI‐Assisted Colonoscopy

**DOI:** 10.1111/1467-9566.70144

**Published:** 2026-01-18

**Authors:** Michael Heinlein

**Affiliations:** ^1^ Institut für Sozialwissenschaftliche Forschung e.V.—ISF Munich Munich Germany

**Keywords:** algorithmic automation, artificial intelligence, clinical gaze, colonoscopy, medical imaging, professional vision, uncertainty, visual practice

## Abstract

This article examines how commercial artificial intelligence (AI) systems are integrated into the visual practice of colonoscopy and how they reshape the clinical gaze as a sociotechnical and situated mode of perception. Based on ethnographic observations of colonoscopies and in‐depth interviews with gastroenterologists, this study analyses the real‐time use of AI‐based pattern recognition systems during diagnostic procedures. Unlike retrospective image analysis in radiology, AI in colonoscopy operates in vivo and in real time, requiring practitioners to engage with algorithmic markings within the unfolding process of examination. The clinical gaze emerges here as a form of professional vision constituted through embodied routines, tacit knowledge, technological infrastructures and institutionalised practices. This article identifies three modes of integrating AI—technology‐driven, experience‐driven and interrupted—that reveal the contingency and multiplicity of human–machine relations in clinical imaging work. Rather than displacing the clinical gaze, AI alters the conditions under which it can be enacted, bringing with it shifting forms of visual selectivity, epistemic authority and new uncertainties. This study contributes to sociological debates on algorithmic medicine and digital automation by showing how AI systems intervene in the practical organisation of medical seeing, highlighting the tensions and adjustments through which contemporary visual practices in healthcare are reconfigured.

## 1 Introduction

Across gastroenterology departments in Germany, AI‐based polyp detection is increasingly becoming part of everyday clinical routines, assisting physicians during colonoscopies in real time. In clinical practice, AI systems are provided as commercial add‐on modules to existing endoscopy platforms and distributed by established endoscopy manufacturers such as Olympus, Medtronic and Fujifilm through hybrid models combining dedicated hardware with ongoing service fees. Based on artificial neural networks (ANNs) trained on large datasets, these systems are designed to detect polyps by highlighting suspicious areas of the colonic mucosa with distinctive on‐screen markers (‘bounding boxes’) during the examination (Haglin et al. 2019; Litjens et al. 2017; Wang et al. 2024). These highlighted areas can then be inspected more closely by the physician.

At first sight, algorithmic pattern recognition appears to complement the clinical gaze, fostering expectations of quicker and more reliable detection of precancerous lesions. Yet, as the analysis will show, clinical practice presents a far more ambivalent picture—one that resonates with recent sociological studies highlighting that the integration of AI systems into medicine is shaped less by clear‐cut gains in efficiency than by persistent tensions and frictions^1^. These include the incomplete and, at times, counterproductive character of automation, as well as the practical, rather than merely ethical or legal, challenges of explainability (Gaglio and Mathieu‐Fritz 2024). Such findings suggest that AI in medicine is less a simple technological fix than one component of a broader sociotechnical practice in which professional routines and algorithmic operations become entangled. Against this backdrop, the integration of AI into colonoscopy cannot be fully understood without considering how it interacts with the forms of professional vision that have long structured diagnostic work.

The concept of *professional vision*, as developed by Goodwin (1994), refers to the embodied, context‐sensitive ways of seeing that are grounded in medical expertise and experience. Professional vision is not reducible to acts of observation but involves interpretation shaped by a range of factors, including formal and tacit knowledge, professional routines and institutionalised practices. In the case of colonoscopy, however, the clinical gaze is not confined to mere acts of *seeing* but also extends to the practices through which internal bodily structures are actively *made visible* in medical work (Burri 2013; Sandfort 2019; van Dijck 2005).

In this context, technology plays a crucial role, as it shapes both the conditions under which visibility is achieved and the ways in which the body's interior is rendered perceptible. This observation is not specific to AI. Modern medical work has long been organised around imaging and visual practices of clinical observation (Burri 2013; van Dijck 2005). From this perspective, colonoscopy is part of the visual regimes that define modern medicine. With the rise of scientific medicine in the 19th century (Foucault 1973), anatomical drawings were gradually replaced by technologically produced images that aspired to ‘mechanical objectivity’ (Daston and Galison 2007). This shift continued with procedures such as X‐ray, ultrasound, CT and MRI, which can be understood as ‘visual localisation techniques’ (Gugerli 1998, 4; my translation) designed to minimise subjectivity at the moment of image production. However, this concerns only the technical form of representation, not the way these images are interpreted in medical work. Without a clinical gaze grounded in knowledge, practice and experience—what Daston and Galison (2007) term ‘trained judgement’—no meaningful interpretation is possible; the technologically produced image and the trained gaze are necessarily entangled in everyday medical practice.

Historically, the relation between image and clinical gaze has been repeatedly reconfigured through the technicisation of medical imaging. Each innovation generated new visual worlds that expanded diagnostic and therapeutic possibilities but only insofar as observation itself was further differentiated and adapted. Radiological images, for example, required physicians to bridge the gap between the visual representation of organic structures and their physical referents, transforming pictures into meaningful diagnoses (Joyce 2008; Sandfort 2019). The introduction of AI extends this trajectory by altering not only how images are produced but also how the clinical gaze is organised. What is at stake, then, is not merely the addition of another observational tool but a shift towards forms of algorithmic automation, where tasks of evaluation and diagnosis are increasingly delegated to machine learning systems.

This shift is particularly consequential in colonoscopy. Technological innovation has long been central to this field, especially in relation to advances in visualisation, light generation and imaging quality (Gangwani et al. 2023). Yet, the deployment of AI marks a qualitative change: For the first time, automated diagnostic assessments are offered *in real time*, during ongoing examinations, requiring immediate engagement by physicians.^2^ Given the recent introduction of AI systems in German gastroenterological departments and the absence of formal guidelines,^3^ this engagement assumes a quasi‐experimental character. Medical research increasingly reflects this, exploring the use of AI ‘in a real‐world setting’ (Levy et al. 2022, 1871) rather than under controlled conditions.^4^

Consistent with this situation, recent studies show that AI in colonoscopy does not automatically yield greater diagnostic certainty; instead, it often introduces moments of ambiguity and uncertainty (Ladabaum et al. 2023; Levy et al. 2022; Taghiakbari et al. 2021). The empirical findings presented in this article suggest that these frictions stem less from the often‐cited opacity of AI, which has been extensively discussed in relation to radiological image interpretation (Lebovitz et al. 2022; Pesapane et al. 2018; Ursin et al. 2022), than from the specific ways in which AI becomes embedded in the situated flow of colonoscopic practice. Opacity remains relevant, but it must be understood within a broader sociotechnical context of medical examination, in which professional vision, routine work practices and technical infrastructures intersect.

Against this backdrop, this article examines how AI‐assisted polyp detection reshapes, and at times, unsettles, the visual practice of colonoscopy, along with its associated forms of knowledge production and professional conduct. More specifically, it asks the following questions: (1) How does the pattern recognition of AI systems intersect with, reinforce or challenge the clinical gaze of gastroenterologists? (2) How does AI become embedded in the situated visual practice of colonoscopy? (3) What are the implications of these dynamics for the epistemic authority of the clinical gaze and for the transformation of visual practices in contemporary medicine? In addressing these questions, this article contributes to the broader discussion on ‘algorithms in practice’ (Christin 2017) and to emerging work on ‘algorithmic medicine’ (Petersen 2018).^5^

In what follows, the analysis traces the often contradictory and ambivalent ways in which AI becomes, or fails to become, integrated into colonoscopic routines. Three ethnographic observations serve as entry points into this process, paving the way for a closer examination of the visual logics of colonoscopy and the practical rationalities through which AI is embedded in medical work. The conclusion then reflects on the broader implications of these dynamics for the reconfiguration of medical visual practices in the age of AI.

## 2 Research Process and Methods

The empirical basis of this article consists of in‐depth qualitative interviews with seven experienced gastroenterologists (two residents, one specialist, one senior physician and three heads of department) and one AI researcher specialised in polyp detection, complemented by eight open participant observations of colonoscopies with and without AI support.^6^ The interviews with physicians and the observations were conducted across four German gastroenterological departments, each using different commercially available AI systems (GI Genius, ENDO‐AID and CAD EYE), which had been in use for periods ranging from a few months to 3 years.^7^ The interview with the AI researcher, by contrast, took place at a research institution working in basic medical image analysis and collaborating with clinical sites. Although the interviews cover all sites and systems, the observational strand differentiates between settings: Four colonoscopies with AI support (ENDO‐AID) were observed in one clinic, and four procedures without AI in another. Three of the interviewed physicians (two residents and one senior physician) were also directly involved in research on AI in endoscopy, offering additional insight into emerging practices and debates within the field.

The research process unfolded iteratively and reflexively (Glaser and Strauss 1967). In this process, hypotheses regarding the incorporation of AI into routine colonoscopy were developed and refined throughout the course of the study. Interviews lasted between 60 and 120 min and followed a semi‐structured, problem‐centred format (Witzel and Reiter 2012). A thematically focused guideline—centred on the use and integration of AI‐supported polyp detection during examinations—served as a basis, whereas the open structure of the interviews allowed participants to introduce their own priorities and share narrative accounts of their experiences with AI. The open participant observations involved consecutive colonoscopy procedures and were conducted by two researchers. They lasted between 30 and 60 min, including both the preparatory and postprocedural phases. During the examinations, we engaged directly with the examining physicians and assisting nursing staff to gain a deeper understanding of the workflows and routines within the examination rooms. Follow‐up observations were designed according to a reflexive research approach, allowing open questions to be further explored and new lines of inquiry emerging during the research process to be systematically pursued. The time in the field extended well beyond interviews and formal observations, as preliminary theses were repeatedly taken back into ongoing conversations with a senior physician and an AI developer over several months and refined in light of these exchanges.

The material was analysed following the principles of the documentary method (Bohnsack 2014), focusing on the implicit, often taken‐for‐granted, knowledge structures and habitual orientations that underlie professional practice. In line with practice‐theoretical approaches (Reckwitz 2002; Schatzki et al. 2001), the analysis treats the handling of AI in clinical settings as shaped by embodied routines, material arrangements and socially shared ways of seeing and knowing. In a first step, the material was coded for themes such as successful and failed integrations, shifting visual routines and negotiations over responsibility in human–AI interaction. In a second step, these findings were interpreted with a view to the orientation frameworks and practical logics guiding physicians' engagement with AI, focusing on how they narrate their experiences, manage uncertainty and disruptions, and position their expertise in relation to algorithmic pattern recognition.

Although the quotations in the analysis indicate the hierarchical position and clinical experience of the respective physicians, the material does not reveal any structural pattern that would systematically shape how AI is integrated into clinical work. Rather, the ways in which AI is taken up appear to emerge situationally within local routines and the immediate demands of the examination. This assessment, however, must be read in light of the small number of interviews and the early‐stage, loosely regulated uses of AI systems examined here.

## 3 *Doing* AI‐Assisted Colonoscopy: Three Observations

To illustrate how AI intervenes in the practice of colonoscopy, this section presents three ethnographic observations. These snapshots show how AI is woven into the situated practice of colonoscopy, in which the clinical gaze is enacted through professional expertise and technologically mediated routines. Although the empirical findings presented below examine these dynamics in greater depth, the aim here is to convey what is at stake in the everyday performance of colonoscopy and how algorithmic pattern recognition becomes part of this practice.

First observation, in a large hospital: In a darkened room, a sedated patient lies on an examination table under surgical lights, positioned on his side. In front of the table, stands a tall device tower with a large monitor mounted at the top. At the head of the table, a nurse regularly injects anaesthetic into the intravenous line placed in the patient's arm and ensures the patient remains properly positioned. Behind the patient, a specialist holds a black, flexible tube, roughly the diameter of a finger—the colonoscope—which is inserted through the anus into the patient's large intestine, the colon. The scope contains several channels: one for insufflating air to expand and smooth the mucosa, one for light transmission, one for flushing with water to flush away residual stool or fluid that might obstruct the physician's view and one for inserting instruments such as biopsy forceps. With her left hand, the physician manipulates a control unit with wheels and buttons to steer the camera and adjust functions at the tip; both the control unit and the tube remain in constant use. As the scope navigates the twisting colon, the physician's body moves in rhythm with the live video feed, adjusting to curves and folds of the mucosa. To her right, a nurse stabilises the patient; to her left, another nurse introduces biopsy forceps into the scope. All eyes focus on the large monitor, where real‐time images of the colon appear. Intermittent green box‐shaped markers—produced by an AI system via a compact processor—flash, vanish or hold steady, highlighting suspicious areas of the mucosa. The specialist guides the forceps towards a section of thickened tissue consistently framed by one of the green markers. ‘Open,’ she says. The nurse activates a lever, and the forceps open. The physician aligns them precisely, then says, ‘Close.’ The nurse shuts the clamp, and the forceps grasp the tissue. With a quick motion, the sample is removed and placed into a specimen container. ‘Nodule, sigmoid,’ the nurse says. ‘Nodule, sigmoid,’ the specialist repeats.

In this observation, AI appears to function in an almost textbook fashion: The AI system marks a conspicuous area of the colonic mucosa, the examining specialist inspects and assesses the marked area, and the tissue is subsequently removed. Everything proceeds smoothly and efficiently. The AI's pattern recognition—trained in advance on countless images of colonic lesions—and the physician's trained gaze complement and support one another with great success. With one important caveat: Although this situation does occur in practice, it is by no means typical. Nor does it reveal much about the actual role that AI plays in relation to the clinical gaze.

Second observation, in the same hospital, during a later colonoscopy in an almost identical setting: The examination has been underway for some time, and the AI is activated and continuously highlights various conspicuous areas of the mucosa. Some of these areas are biopsied; others are not, with the examining specialist commenting each time, ‘That's nothing.’ She explains to us that AI is very helpful, especially when concentration starts to wane after several procedures: ‘It's like having 10 extra eyes!’ But just a few minutes later, the physician reaches behind her to the equipment tower, presses a button and switches off the AI: ‘These bounding boxes are getting on my nerves.’ The examination continues without it.

Third observation: In another examination, the AI also marks a suspicious area of the colonic mucosa. The examining resident and a nurse discuss whether it is really a polyp or not. Both are uncertain, despite the AI's marking. After a brief discussion, they decide to inject saline beneath the area, hoping this will make the contours of a potential polyp more clearly visible. The injection proves difficult and only succeeds after several attempts. Meanwhile, the AI continues to mark both the area under examination and the syringe in use; green boxes flicker continuously on the screen and disappear again. The procedure yields no clear results but leads the physician and the nurse to suspect that it is likely not a polyp. They continue to observe the area and debate whether to perform a biopsy. A specialist is called in to assess the situation. She looks at the screen and concludes, ‘No, that's not a polyp.’ The area is not sampled, and the examination proceeds.

## 4 Logics of Seeing: Colonoscopic Visual Practices

These empirical snapshots provide a starting point for analysing the clinical gaze in colonoscopy, not as a fixed attribute of medical expertise, but as a visual practice that is constantly enacted, negotiated and increasingly mediated by digital technologies. Drawing on Burri's (2012) concept of ‘visual logic’, the visual practice of colonoscopy can be understood as shaped by specific ways of seeing, interpreting and acting on visual information. These ways are not simply dictated by the technical properties of imaging devices but emerge through the social and material arrangements in which such technologies are embedded. Following Timmermans and Berg's (2003) notion of ‘technology‐in‐practice’, colonoscopy emerges as a dynamic field in which professional vision, bodily routines and technologies become entangled in the ongoing production of medical knowledge. The visibility of anatomical structures during colonoscopy is *co‐produced* through the positioning of the patient's body, the manipulation of the endoscopic device, the resolution and contrast of the imaging system, camera technology, light source and, increasingly, through algorithmically generated markers that highlight areas of presumed clinical relevance. In this sense, the clinical gaze in colonoscopy can be understood through the lens of ‘emergent visualities’ (Rose and Tolia‐Kelly 2016, 2): It is constituted within an embodied, experience‐ and knowledge‐based, as well as technically and materially mediated, social practice and is linked to the images of the colonic mucosa that are made visible and open to interpretation in that context.

What resonates in the observations presented above, and becomes evident across the empirical material more broadly, are two logics of professional seeing that shape the visual practice of colonoscopy. Each reflects distinct epistemic practices, yet they remain deeply intertwined in action. The first is *focused seeing*, which involves the visual assessment of a specific section of tissue. This is not merely a solitary act of looking but a process of integrating multiple individual gazes and interpretations, often shaped through both verbal and nonverbal communication among the clinical team. The second is *contextual seeing*, which refers to perceiving the colon as a navigable spatial environment. This requires physicians to read and understand the visible space, to orient themselves within it and to grasp what the colon is doing at any given moment—whether it is calm or contracting—and what is happening inside it, such as the movement of fluids, stool or bubbles.^8^ These logics interact continuously in practice and form the basis of clinical navigation and judgement during colonoscopy. To shift between them, experienced practitioners perform what one physician referred to as a ‘circular gaze’ (Specialist, l. 47) or, as another put it, a ‘U‐shaped pendulum movement, looking left and right, always scanning the edges [of the visible colon wall]’ (Resident 2, ll. 191–192).^9^ When something suspicious appears, the gaze may come to rest on a particular area of the mucosa for closer inspection.

Both focused and contextual seeing are acquired through sustained and regular practice during the clinical training of gastroenterologists. Experienced physicians accompany trainees over several months during colonoscopy procedures. In addition, the accuracy of the trainees is continuously evaluated by histologically assessing the clinical relevance of the detected and removed polyps. Visual skills thus emerge directly within the visual practice of colonoscopy and are tightly coupled to the epistemic and practical knowledge base of the discipline.^10^

## 5 Inscriptions of Seeing: AI‐Based Polyp Detection

With the advent of AI‐based detection systems, however, these long‐standing forms of colonoscopic visual practice are confronted with new logics of vision encoded in algorithmic design. To understand the frictions observed in practice, it is therefore necessary to examine how particular ways of seeing are inscribed into ANNs and embedded in the functioning of AI technologies.^11^ The development of ANNs for the analysis of medical images began as early as the 1990s (Fazal et al. 2018; Litjens et al. 2017). However, it was not until the 2010s, with significant advances in machine learning techniques and computational power, that this form of AI achieved a breakthrough in colonoscopy (Wittenberg and Raithel 2020). Clinical products such as Medtronic's GI Genius, Fujifilm's CAD EYE, PENTAX's DISCOVERY and Olympus's ENDO‐AID have been commercially available since 2019. The ANNs used in these systems are pretrained on vast datasets of colonoscopy images featuring histologically verified abnormalities such as tumours, polyps or inflammations. Before training, qualified personnel annotate these abnormalities by outlining the relevant areas (Wang et al. 2024), making visible the patterns the ANN is expected to recognise in practice. The model—usually a convolutional neural network—is then trained in a supervised process with annotated data that already contain the correct image interpretations. This allows the AI to evaluate whether its predictions match the known outcomes and iteratively improve its accuracy. Throughout the training process, the model's performance is regularly validated to ensure that it does not simply memorise the training data but also performs reliably on new, previously unseen images. After training, the model undergoes evaluation on a separate test dataset to assess its generalisation capabilities and fine‐tune its performance. Only after successfully completing these phases and demonstrating sufficient reliability and accuracy in clinical trials is the model approved for real‐world clinical use.

As discussed above, technically generated medical images are not naturalistic representations of the body's internal structures. Rather, they produce visual worlds that place specific demands on the clinical gaze and, in doing so, reshape it.^12^ This applies all the more to technologies that automate the analysis of medical images. These technologies are not neutral tools; their functioning is shaped by ‘certain interpretive schemes (rules reflecting knowledge of the work being automated), certain facilities (resources to accomplish that work) and certain norms (rules that define the organisationally sanctioned way of executing that work)’ (Orlikowski 1992, 410), intentionally or inadvertently inscribed during their development and training. In the case of the ANNs used in colonoscopy, the training process reveals that the vision they enact is *formalisable*, *uniform* and *independent of situational contexts*, operating as an isolated act of localisation detached from the embodied and situationally embedded character of the clinical gaze (Anichini et al. 2024).

These inscribed forms of algorithmic vision present a significant challenge to everyday medical practice. The visual work of colonoscopy follows its own logic and must, in real time and under situational conditions, actively engage with AI‐based pattern recognition—an input that remains externally encoded yet intervenes immediately and materially in the ongoing practice. This interaction harbours a distinct ‘potential for breakdown’ (Akrich 1992, 207) in the use of AI: Although the ANNs used for colonoscopy are trained on vast datasets of annotated images, embodying an aggregated clinical gaze that has encountered more colonic lesions than any individual practitioner, this inscribed form of vision fundamentally differs from the professional gaze of physicians. Although what becomes visible—and thus actionable—is increasingly shaped by training data and algorithmic design, the professional gaze extends beyond this, involving ways of seeing that cannot be reduced to the mere registration of formal data. The following analysis indicates that the tensions at these intersections are not accidental. Rather, they reflect a *structural mismatch* between practice‐based ways of seeing/knowing and the operational rationality inscribed into AI systems.

## 6 Visual Practices of Colonoscopy and the (Non)Integration of Artificial Intelligence

In our empirical material, we observe distinct dynamics that each characterise a specific mode of engagement with algorithmic pattern recognition in the visual practice of colonoscopy. First, a *technology‐driven integration*, in which the visual practices of colonoscopy are centred on the operations of AI, leading to a technologically guided transformation of the clinical gaze. Second, an *experience‐driven integration*, where the technological operations of AI are subordinated to the experienced gaze of physicians. Here, AI becomes embedded in such a way that the clinical gaze changes but remains dominant and largely sovereign. Third, an *interrupted integration*, in which AI unsettles the clinical gaze to the point that the ongoing examination is disrupted or even comes to a complete halt.

These dynamics should not be understood as fixed categories that map neatly onto different groups of practitioners. Rather, they represent empirically grounded practical rationalities that could be reconstructed across interviews and observations. The technology‐driven and the experience‐driven modes express professional attempts to stabilise the use of AI under conditions in which its role, value and reliability are still unsettled. Both can be read as situated responses to the uncertainty of how algorithmic pattern recognition should be handled in the flow of colonoscopic work. The interrupted mode, by contrast, points to moments in which a technical rationality asserts itself—one shaped by the contingencies and unpredictabilities of AI systems that resist immediate professional integration (Heinlein 2024; Heinlein and Huchler 2023).

Crucially, these rationalities do not appear in isolation. They overlap, intermingle and, at times, contradict one another within the same examination or even within a single practitioner's account. Their coexistence reflects not stable orientations but the quasi‐experimental character of AI use in contemporary endoscopy: Physicians are testing, adjusting and recalibrating how to work with algorithmic vision in real time, without established routines or clinical guidelines to rely on. Against this backdrop, the three modes are best understood as analytical reconstructions that make visible the shifting attempts to align algorithmic operations with the situated logics of the clinical gaze.

Similar dynamics can be observed in other areas of medicine that are integrating AI, as shown by Avnoon and Oliver's (2023) discourse‐analytical study of radiology. They describe how medical professionals engage in a ‘rhetorical dance’ of integration, subordination, resistance and assimilation in response to AI, employing strategies that help maintain professional authority. Although their analysis focuses on the negotiation of professional boundaries, this study shifts attention to the material and sociotechnical reconfigurations of medical vision in everyday diagnostic work—bringing into view the *practical dance* between gastroenterologists and AI systems as it unfolds in real‐time, embodied interaction.

### 6.1 Technology‐Driven Integration

In the technology‐driven mode, AI is given a central role in guiding diagnostic attention, with physicians routinely activating the system and aligning their gaze with its markers—an orientation that is illustrated most clearly in the first observational snapshot, where the examination unfolds along the logic of the algorithmic display. Although not yet regarded as a ‘gamechanger’ (Head 3, l. 203), some interviewees anticipate that within five to 10 years, AI may operate with less reliance on the trained clinical gaze. AI, they contend, will then no longer require the physician's gaze—but this shift would not render their role obsolete. Although AI may eventually operate independently of the trained clinical gaze, it will still depend on physicians' practical actions to function effectively. As several interviewees pointed out, tasks such as manoeuvring the colonoscope, positioning patients and ensuring optimal visual conditions (i.e., ensuring smooth mucosal surfaces, minimising peristalsis and flushing away residual fluids) remain essential for producing the image data required for accurate polyp detection.

Accordingly, the current use of AI is framed as a transitional phase in which the still immature technological performance of AI is perceived not only as a practical challenge but also, at times, as a source of *uncertainty* or even as a *threat* to the authority of the trained clinical gaze. Three dynamics, in particular, illustrate how this uncertainty takes shape:

First, in the interviews, the adenoma detection rate (ADR) of AI—its ability to identify medically relevant changes in the colon mucosa—is frequently described as superior to the human gaze. This includes detecting even the smallest mucosal changes that, if perceptible to the human eye at all, would previously have been left in situ because they were not considered clinically significant. Now, however, these changes are systematically biopsied or removed.^13^ The resulting additional workload is consciously accepted and justified by the perceived imperative to act upon AI‐detected lesions: ‘Because this AI detects lesions that we don't see, it takes us longer now, because we have to biopsy all of them’ (Head 1, ll. 83–84). The promise of reducing future workload is invoked—’You're saving yourself from having to remove that polyp in five or 10 years’ (Senior, ll. 150–151)—yet physicians simultaneously note that acting on every marker introduces practical and diagnostic uncertainty: How small is too small, and when is action clinically warranted?

Second, the ‘circular gaze’ (Specialist, l. 47) practised in colonoscopy—the methodical scanning of the mucosa—has begun to lose its taken‐for‐granted status. Interviewees increasingly frame it as something that must be defended: ‘I have to say, I've been performing colonoscopies for quite a long time, and I've done it for about 6 years without AI, so it's just trained into me to still look in circular ways’ (Specialist, ll. 43–45). The small word ‘still’ marks a subtle shift: An embodied routine once unquestioned now appears in tension with the technologically mediated expectation that following AI markers may suffice. This reveals a deeper contradiction: AI scans rapidly, invisibly and without embodied orientation; physicians, by contrast, enact vision through eye, head and body movements. The mismatch between these modes of seeing generates uncertainty about what counts as adequate inspection and whether embodied routines risk becoming obsolete.

Third, this tension becomes particularly evident in situations where developing a trained gaze with AI is perceived as a laborious process, despite a clear commitment to a technology‐driven use of AI: ‘Young people just stare into the centre already when using AI, thinking, well, eventually when the AI picks something up, a box will appear anyway’ (Resident 2, ll. 186–187). The circular gaze is still regarded as essential and professionally significant, as it apprehends the colon spatially through sweeping but methodical visual movements and provides occasions for a focused examination of conspicuous lesions. ‘Staring into the centre’, which amounts to passively waiting for AI‐generated boxes to appear, is not perceived as professional or goal‐oriented.

Taken together, these moments show that the technology‐driven mode of AI integration is neither a stable path towards an algorithmically automated clinical gaze nor free of internal tensions. Even those who consistently align their attention with AI markers emphasise that core elements of visual practice—above all, the circular gaze and the spatial reading of the colon—cannot be abandoned without risking diagnostic quality, revealing a persistent unease about what is lost when professional vision is reshaped too strongly around algorithmic cues. As the following section shows, similar ambivalences also characterise the experience‐driven mode: Even when AI is explicitly subordinated to clinical expertise, the interplay between algorithmic detection and the trained gaze remains marked by uncertainty over how medical seeing should evolve in practice.

### 6.2 Experience‐Driven Integration

In the dynamic of experience‐driven integration, algorithmic pattern recognition is not seen as superior to the trained clinical gaze. Instead, AI is evaluated in a sober and pragmatic manner: There is ‘potential for improvement’ (Head 2, l. 284), but it is expected to remain subordinate to clinical experience and not to replace the physician's gaze. From this perspective, AI is valued primarily as a compensatory tool: ‘Its strength probably lies in the fact that it helps less experienced examiners or even when you're at the end of a long day and can't really concentrate anymore. Then, you really appreciate the support from AI—that's definitely something you can imagine well’ (Head 2, ll. 48–51). At the same time, several interviewees noted that medical trainees are expected to refrain from using AI at certain stages in order to develop the experiential grounding required for a trained clinical gaze. This expectation sits uneasily alongside narratives that emphasise AI's usefulness precisely for less experienced practitioners. The tension illustrates how the very process of gaining experience becomes more complex under algorithmic conditions and shows that even within a single mode of integration, practitioners articulate contradictory orientations towards the role of AI in learning and practice. Nonetheless, whether AI is used at all, and how its markings are interpreted and acted upon, remains a matter of ‘individual examiner judgement’ (Head 2, l. 68).

When AI is used, its operations are clearly subordinated to the experienced clinical gaze. This becomes visible, first, in *image production*: As several physicians noted, the conditions under which AI performs well are essentially those that also support optimal human vision. Unlike in the technology‐driven mode, no additional manoeuvres are undertaken solely to generate ideal input for the system. Second, this subordination extends to *image interpretation*. Physicians emphasise that every AI‐marked area requires critical human review and that the system's relevance assignments must be weighed against their own knowledge and experience: ‘A colonoscopy takes about half an hour, and if the AI is running the whole time, it makes thousands of measurements or suggestions. And, yes, many of those you just have to quickly disregard’ (Resident 1, ll. 177–180). In this sense, the clinical gaze becomes a human observation of a technical observation, assessing machine‐generated cues in real time through experience and professional judgement.

Even within the technology‐driven mode, physicians may ultimately challenge AI‐supported suggestions—as the third observation in the introductory snapshots illustrates—but doing so typically requires extended deliberation and the negotiation of doubt within the team. By contrast, in the experience‐driven mode, such divergence is enacted far more readily. Here, it is the physician's accumulated expertise that authorises departures from AI assessments. One interviewee described a case in which the system not only flagged suspicious areas but also assigned probability scores indicating whether a lesion was malignant or benign: ‘And AI can of course distinguish whether it's a polyp likely to become cancerous or a so‐called hyperplastic polyp, which basically doesn't tend to develop into cancer and could theoretically be left alone. But we've had discrepancies where I said, no, we'll remove it anyway, even though the AI said it's benign—and then histology confirmed that it was indeed something where you'd say, okay, good thing we removed it’ (Head 2, ll. 52–59). In these situations, the trained clinical gaze retains clear primacy. Unlike in the technology‐driven mode—where even clinically irrelevant AI‐marked polyps are routinely removed—the decision to override the system is framed as part of professional judgement rather than an exception. AI becomes one element within the visual practice of colonoscopy, but not its guiding principle.

This raises the broader and widely discussed issue of responsibility in AI‐supported medical examinations (Grote and Berens 2020; Krügel et al. 2025). Formally, both the technology‐driven and the experience‐driven mode arrive at the same conclusion: Responsibility for the procedure and all diagnostic decisions remains with the physician, not least because no guidelines currently regulate AI use in colonoscopy. Yet this shared conclusion rests on very different logics. In the technology‐driven mode, the perceived superiority of AI leads to a systematic increase in biopsies, framed as greater thoroughness and as an unproblematic extension of clinical responsibility. Here, acting on AI‐generated cues appears self‐evident. In the experience‐driven mode, the same conclusion is reached for very different reasons. Responsibility is anchored in professional judgement and cannot be delegated to the system: ‘It's a learning process. AI is like a very, very experienced colleague you hope you can rely on—but you still have to think it through and check for yourself, right? You're responsible. You can't just say the AI made a mistake. So, you still have to learn this yourself’ (Senior, ll. 270–274). From this perspective, AI neither substitutes nor simplifies the continual work of sustaining the clinical gaze. The often‐invoked metaphor of an ‘experienced colleague’ highlights, rather than dissolves, the tension between trained human vision and algorithmic judgement. This tension grows out of different forms of experience—clinical expertise, the ongoing work of learning to use AI and the experience encoded in the system itself—but across interviews physicians emphasised that responsibility for diagnosis must remain with the practitioner.

### 6.3 Interrupted Integration

Although the technology‐driven and experience‐driven scenarios both seek to functionally incorporate AI into the visual practice of colonoscopy, a further dynamic becomes visible—one that can be described as *interrupted integration*. It emerges in situations where AI unsettles the clinical gaze or becomes disruptive to such an extent that the examination is briefly halted or must be actively restored. In these moments, the system appears as a resistant technical object (Latour 2000), eluding attempts—whether technologically or experientially oriented—to stabilise its function in practice. Because the AI can be switched on or off at any time, this resistance is resolved pragmatically: As in the second observational snapshot presented at the beginning of this article, the system is deactivated—temporarily or permanently—so that the procedure can continue without disturbance.

This dynamic manifests in several ways. One example is when the AI *overwhelms* the clinical gaze by confronting the examining physician with an interface that demands rapid visual processing and immediate interpretation of a variety of visual and auditory cues. One physician described the cognitive overload in this way: ‘There are so many factors—alarms, colours, sounds, some kind of interface—and I don't understand it. Curves and bars going up and down… it's just not being accepted’ (Resident 2, ll. 194–196). Second, AI is perceived as a *hindrance* to the clinical gaze when its visual markers obstruct the physician's view during critical moments of intervention: ‘I notice that you quickly get… well, annoyed, or… you just find it distracting, these markings. And, for example, when you find a polyp and want to remove it, the marking has to disappear. You've already found it, and then the marking is just in the way, right?’ (Resident 1, ll. 209–213). Both examples point to future requirements for design and training: Interfaces need to be better aligned with physicians' cognitive capacities, and AI should be trained to recognise polyp removal so that markers temporarily disappear during intervention.

By contrast, a third form of interruption is linked to the inherent *unpredictability* and *contingency* of AI (Heinlein 2024). In such cases, AI unsettles the clinical gaze not by producing cognitive overload or errors but by introducing a form of uncertainty rooted in its own logic of pattern recognition. As one physician noted, ‘We're repeatedly surprised when the AI picks something up, and then you look’ (Head 3, ll. 52–53). Here, the discrepancy between algorithmically highlighted areas and the physician's expectations of diagnostically relevant regions triggers a moment of uncertainty. It is not merely a question of confirming or dismissing a highlighted marker upon closer inspection. Rather, the AI destabilises perceptual certainty by producing genuinely surprising results—outcomes that are unexpected, difficult to interpret and that introduce new forms of clinical ambiguity. In the observations, such moments of uncertainty occurred repeatedly and occasionally became the subject of brief discussions among the medical staff—or were accompanied by spontaneous remarks such as ‘Huh, what's the AI doing now?’.

Crucially, this indeterminacy is not to be confused with imaging artefacts arising from radiographic or digital imaging technologies. The uncertainty here stems from the fact that AI is credited with a form of pattern recognition that is perceived as equivalent to expert human vision. AI is experienced as a support system whose surprising markings might, in principle, be *valid*. In such situations, AI is not turned off; the examination continues, but under the shadow of an unexpected cue that must be critically reviewed—yet cannot be fully ignored.

## 7 Conclusion: Reconfiguring the Clinical Gaze Under Algorithmic Conditions

Although the scope of the findings presented here is necessarily limited, the early phase of AI adoption in colonoscopy is analytically revealing: It shows that algorithmic automation does not unfold in a linear or uniform manner and that the clinical gaze does not simply recede but remains a central site in which the practical consequences of AI integration are negotiated.

AI's pattern recognition does not simply enhance diagnostic certainty but reshapes the clinical gaze in uneven and ambivalent ways. In the mode of technology‐driven integration, algorithmic markers can come to dominate the course of action, at times prompting the removal of every indicated lesion. In the mode of experience‐driven integration, by contrast, such cues are situated within the authority of professional expertise and embodied judgement and may be selectively followed or set aside. Both modes involve an assessment of relevance, yet they weight the relationship between algorithmic signal and clinical gaze differently. In practice, the balance remains unstable, and AI may disrupt the visual flow of examination to the point of hesitation, reorientation or even temporary suspension.

However, physicians do not adopt fixed roles as enthusiasts, sceptics or pragmatists; instead, they navigate shifting constellations in which different logics of seeing intersect. Integration, in this sense, is a dynamic process in which the clinical gaze itself becomes a site of experimentation. This openness should not be reduced to questions of habituation nor assumed to disappear with further technical refinement. Algorithmic automation does not simply occur but is *enacted in practice* (Heinlein and Huchler 2023), requiring constant alignment and correction between algorithmic vision and professional seeing.

Unlike radiology, where algorithmic assessments can often be reviewed asynchronously (Anichini and Kotras 2024), colonoscopy confronts physicians with AI‐generated cues in real time, demanding that they incorporate algorithmic signals into their visual work within seconds. This immediacy turns the management of attention and judgement into a distinctive challenge of AI‐assisted colonoscopy. At the same time, the use of ANNs transforms not only what is seen but also the very conditions under which things become visible. Algorithmic markers cut into the visual field itself, inscribing interpretive significance that traditionally depended on human perception and tacit expertise. They do not merely accentuate pre‐existing features; they actively redirect visual attention and recalibrate what counts as diagnostically relevant. In this sense, algorithms alter ‘the ways the visual signs are composed in an image’ (Burri 2012, 51), reshaping the grammar of the clinical gaze itself.

As a consequence, epistemic authority no longer rests solely with the physician but is redistributed across hybrid constellations where human and algorithmic judgements are intertwined. AI systems inscribe their own logics of vision into the examination, which do not simply mirror the conventions of clinical practice (Anichini et al. 2024). The empirical findings presented in this article suggest that physicians respond to this by developing reflexive strategies—deciding when to trust, when to doubt and when to override algorithmic suggestions. From this perspective, the clinical gaze does not vanish but becomes a site of adaptation and recalibration, marked by new forms of vigilance and responsibility.

These dynamics are further complicated when considering the cultural scripts of the ‘endoscopic gaze’, as van Dijck (2001) argues. The technological ability to visualise the body's interior without invasive surgery has long been accompanied by the cultural fantasy of the transparent and fully knowable body. Endoscopic procedures and now AI‐assisted image analysis extend this fantasy by suggesting a seamless and objective view into the body's interior, reinforcing the myth of a body without borders—fully accessible to vision, fully open to intervention, sustained by the promise that it can be made intelligible ‘through the lens of the deep learning model’ (Amoore 2023, 31). Yet, this idealised vision remains partial and selective, shaped by the capabilities and constraints of both the clinical gaze and the machine vision designed to support it.

This, in turn, points to the importance of investigating how *uncertainty* emerges as a constitutive feature of algorithmic medicine, rather than a temporary by‐product of technological immaturity. In fact, medicine has never rid itself of uncertainty in clinical practice—despite, or perhaps precisely because of, its efforts at standardisation and technicisation (Fox 2000; Timmermans and Angell 2001). The use of AI does not suspend this condition; instead, the clinical gaze is confronted with new uncertainties that arise from the ways algorithmic pattern recognition intervenes in colonoscopic visual practices. In AI‐assisted colonoscopy, it is not the bounding boxes themselves that are ambiguous but their interplay with embodied routines and situated knowledge through which physicians assess lesions; such interplay can interrupt the visual flow of examination and produce moments of doubt or hesitation. What is reconfigured under algorithmic conditions is not uncertainty itself but the shifting nexus of *technicisation* and *medical practice*. Moving beyond the promises of ‘technological “quick fix” solutions that seek to manage uncertainty’ (Mackintosh and Armstrong 2020, 2) is therefore essential for understanding how algorithmic medicine generates new indeterminacies and what practical demands these place on the clinical gaze. In this light, the clinical gaze appears less as a residue of predigital medicine, reducible to algorithmic automation. Rather, it constitutes a site where the transformations of algorithmic medicine are consistently worked through.

## Author Contributions

**Michael Heinlein:** conceptualization, data curation, formal analysis, funding acquisition, investigation, methodology, validation, writing – original draft, writing – review and editing.

## Funding

This work was supported by the Deutsche Forschungsgemeinschaft (DFG) (project number 523799112).

## Ethics Statement

All interviews and observations were conducted in 2024 as part of the DFG‐funded project ‘Artificial Vision at Work’. Participants were fully informed about the research questions, methods and objectives of the project and provided their consent to take part in the study. The author is committed to the Guidelines for Safeguarding Good Research Practice issued by the Deutsche Forschungsgemeinschaft (DFG) and to the Code of Ethics of the Deutsche Gesellschaft für Soziologie (DGS).

## Conflicts of Interest

The author declares no conflicts of interest.

## Data Availability

Research data are not shared.

## Endnotes

^1^
See, for instance, the 2024 Special Issue of the journal *Réseaux* on the practical integration of AI in clinical practice (https://shs.cairn.info/journal‐reseaux‐2024‐6?lang=en).

^2^
This is evident in a rapidly expanding, predominantly experimental research landscape, where studies on AI‐assisted colonoscopy address not only technical performance but also clinical implications such as shifts in physicians' gaze patterns and examiner fatigue.

^3^
In Germany, the ‘Deutsche Gesellschaft für Gastroenterologie, Verdauungs‐ und Stoffwechselkrankheiten’ (German Society for Gastroenterology, Digestive and Metabolic Diseases) issues clinical guidelines for endoscopy. In the consultation version of the current guideline from January 2025, it is stated with regard to AI that ‘a conclusive recommendation for its routine use in screening endoscopy appears premature at this time, given the unclear clinical relevance of the increased detection of small adenomas’ (Denzer et al. 2025, 212; my translation).

^4^
Findings in this area primarily concern adenoma detection rates, which do not improve with the use of AI as expected or in some cases even decline (Ladabaum et al. 2023; Levy et al. 2022; Taghiakbari et al. 2021).

^5^
Although this article does not primarily examine concerns about potential deskilling (cf. Budzyń et al. 2025), the empirical material indicates that physicians interpret AI less as a threat to expertise than as contributing to a gradual recomposition of competencies.

^6^
The empirical material derives from the research project ‘Artificial Vision at Work’, which is funded by the German Research Foundation (DFG) as part of the interdisciplinary priority programme ‘Digitalisation of Working Worlds’ and runs from October 2023 to September 2026.

^7^
Although specific cost data were not disclosed, interviewees noted that acquiring such systems is economically viable for hospitals but considerably less attractive for private practices due to lower procedure volumes.

^8^
In an eye‐tracking study of chest CT scans, Drew et al. (2013) distinguish between ‘scanners’, who survey entire axial slices, and ‘drillers’, who focus on a quadrant and scroll rapidly through successive layers—findings that are closely in line with the heterogeneous visual strategies observed in this study.

^9^
All quotes are taken from interviews with German gastroenterologists and have been translated into English by the author.

^10^
For related accounts of how visual expertise is practically acquired, see Joyce (2008) and Prasad (2005), who examine the interpretation of MRI imagery.

^11^
The development of AI‐based polyp detection is traced primarily through insights gained from interviews with the researchers involved.

^12^
As Joyce (2006) illustrates with the example of MRI, the reverse is also true: The practice of providing both images and their numerical data was discontinued as soon as MRI entered clinical settings, where radiologists insisted on image‐only outputs.

^13^
Studies on AI‐assisted colonoscopy primarily report improvements in detection rates under experimental or semi‐controlled conditions. However, systematic data on the proportion of biopsies that specifically confirm AI‐indicated lesions remain scarce, making it difficult to assess how often algorithmically highlighted findings are clinically validated. This lack of granularity is itself indicative of the uncertainties that accompany the early practical integration of AI into colonoscopic work.

## References

[shil70144-bib-0001] Akrich, M. 1992. “The De‐Scription of Technical Objects.” In Shaping Technology/Building Society: Studies in Sociotechnical Change, edited by W. E. Bijker and J. Law , 205–224. MIT Press.

[shil70144-bib-0002] Amoore, L. 2023. “Machine Learning Political Orders.” Review of International Studies 49, no. 1: 20–36. 10.1017/s0260210522000031.

[shil70144-bib-0003] Anichini, G. , and B. Kotras . 2024. “Trained Judgements Artificial Intelligence, Epistemic Tensions and the Production of Scientific Objectivity.” Science, Technology & Human Values. 10.1177/01622439241262854.

[shil70144-bib-0004] Anichini, G. , C. Natali , and F. Cabitza . 2024. “Invisible to Machines: Designing AI That Supports Vision Work in Radiology.” Computer Supported Cooperative Work 33, no. 4: 993–1036. 10.1007/s10606-024-09491-0.

[shil70144-bib-0005] Avnoon, N. , and A. L. Oliver . 2023. “Nothing New Under the Sun: Medical Professional Maintenance in the Face of Artificial Intelligence’s Disruption.” Big Data & Society 10, no. 2: 20539517231210269. 10.1177/20539517231210269.

[shil70144-bib-0006] Bohnsack, R. 2014. “Documentary Method.” In SAGE Handbook of Analyzing Qualitative Data, edited by U. Flick , 217–233. SAGE.

[shil70144-bib-0007] Budzyń, K. , M. Romańczyk , D. Kitala , et al. 2025. “Endoscopist Deskilling Risk After Exposure to Artificial Intelligence in Colonoscopy: A Multicentre, Observational Study.” Lancet Gastroenterology & Hepatology 10, no. 10: 896–903. 10.1016/s2468-1253(25)00133-5. 40816301

[shil70144-bib-0008] Burri, R. V. 2012. “Visual Rationalities: Towards a Sociology of Images.” Current Sociology 60, no. 1: 45–60. 10.1177/0011392111426647.

[shil70144-bib-0009] Burri, R. V. 2013. “Visual Power in Action: Digital Images and the Shaping of Medical Practices.” Science as Culture 22, no. 3: 367–387. 10.1080/09505431.2013.768223.

[shil70144-bib-0011] Christin, A. 2017. “Algorithms in Practice: Comparing Web Journalism and Criminal Justice.” Big Data & Society 4, no. 2: 205395171771885. 10.1177/2053951717718855.

[shil70144-bib-0012] Daston, L. , and P. Galison . 2007. Objectivity. Zone Books.

[shil70144-bib-0013] Denzer, U. , A. Arlt , A. Eickhoff , U. Rosien , A. A. Röth , and R. Jakobs . 2025. S2k‐Leitlinie Qualitätsanforderungen in der gastrointestinalen Endoskopie der Deutschen Gesellschaft für Gastroenterologie, Verdauungs‐ und Stoffwechselkrankheiten (DGVS). Version 2.0 – Januar 2025 – AWMF Register Nr. 021–022. https://www.dgvs.de/wp‐content/uploads/2023/04/S2k‐LL‐Qualitaetsanforderungen‐Endoskopie‐v2.0_Konsultationsfassung_Langfassung.pdf. 10.1055/a-2822-0271 42624094

[shil70144-bib-0014] Drew, T. , M. L. H. Vo , A. Olwal , F. Jacobson , S. E. Seltzer , and J. M. Wolfe . 2013. “Scanners and Drillers: Characterizing Expert Visual Search Through Volumetric Images.” Journal of Vision 13, no. 10: 1–13. 10.1167/13.10.3. PMC3736761 23922445

[shil70144-bib-0015] Fazal, M. I. , E. P. Muhammed , T. Jamie , and Y. Gupta . 2018. “The Past, Present and Future Role of Artificial Intelligence in Imaging.” European Journal of Radiology 105: 246–250. 10.1016/j.ejrad.2018.06.020. 30017288

[shil70144-bib-0016] Foucault, M. 1973. The Birth of the Clinic: An Archaeology of Medical Perception. Routledge.

[shil70144-bib-0017] Fox, R. 2000. “Medical Uncertainty Revisited.” In Handbook of Social Studies in Health and Medicine, edited by G. L. Albrecht , R. Fitzpatrick , and S. C. Scrimshaw , 409–425. SAGE.

[shil70144-bib-0018] Gaglio, G. , and A. Mathieu‐Fritz . 2024. “AI, Medicine and the Social Sciences: For a New Perspective.” Réseaux 248: I–XVII. 10.3917/res.248.0011.

[shil70144-bib-0019] Gangwani, M. K. , A. Aziz , D. S. Dahiya , M. Nawras , M. Aziz , and S. Inamdar . 2023. “History of Colonoscopy and Technological Advances: A Narrative Review.” Translational Gastroenterology and Hepatology 8, no. 18: 18. 10.21037/tgh-23-4. 37197258 PMC10184027

[shil70144-bib-0020] Glaser, B. , and A. Strauss . 1967. The Discovery of Grounded Theory. Strategies for Qualitative Research. Aldine Publishing Company.

[shil70144-bib-0021] Goodwin, C. 1994. “Professional Vision.” American Anthropologist 96, no. 3: 606–633. 10.1525/aa.1994.96.3.02a00100.

[shil70144-bib-0022] Grote, T. , and P. Berens . 2020. “On the Ethics of Algorithmic Decision‐Making in Healthcare.” Journal of Medical Ethics 46, no. 3: 205–211. 10.1136/medethics-2019-105586. 31748206 PMC7042960

[shil70144-bib-0023] Gugerli, D. 1998. Die Automatisierung des ärztlichen Blicks. (Post)moderne Visualisierungstechniken am menschlichen Körper. ETH Zürich.

[shil70144-bib-0024] Haglin, J. M. , G. Jimenez , and A. E. M. Eltorai . 2019. “Artificial Neural Networks in Medicine.” Health Technology 9, no. 1: 1–6. 10.1007/s12553-018-0244-4.

[shil70144-bib-0025] Heinlein, M. 2024. “Artificial Intelligence and Contingency: A Practice‐Theoretical Perspective.” In Artificial Intelligence in Society. Social, Political and Cultural Implications of a Technological Innovation, edited by M. Heinlein and N. Huchler , 15–50. Springer.

[shil70144-bib-0026] Heinlein, M. , and N. Huchler . 2023. “Artificial Intelligence in the Practice of Work: A New Way of Standardising or Maintaining Complexity?” Work Organisation, Labour & Globalisation 17, no. 1: 34–60. 10.13169/workorgalaboglob.17.1.0034.

[shil70144-bib-0027] Joyce, K. A. 2006. “From Numbers to Pictures: The Development of Magnetic Resonance Imaging and the Visual Turn in Medicine.” Science as Culture 15, no. 1: 1–22. 10.1080/09505430600639322.

[shil70144-bib-0028] Joyce, K. A. 2008. Magnetic Appeal: MRI and the Myth of Transparency. Cornell University Press.

[shil70144-bib-0029] Krügel, S. , J. Ammeling , M. Aubreville , A. Fritz , A. Kießig , and M. Uhl . 2025. “Perceived Responsibility in AI‐supported Medicine.” AI & Society 40, no. 3: 1485–1495. 10.1007/s00146-024-01972-6.

[shil70144-bib-0030] Ladabaum, U. , J. Shepard , Y. Weng , M. Desai , S. J. Singer , and A. Mannalithara . 2023. “Computer‐Aided Detection of Polyps Does Not Improve Colonoscopist Performance in a Pragmatic Implementation Trial.” Gastroenterology 164, no. 3: 481–483. 10.1053/j.gastro.2022.12.004. 36528131 PMC11521095

[shil70144-bib-0031] Latour, B. 2000. “When Things Strike Back: A Possible Contribution of ‘Science Studies’ to the Social Sciences.” British Journal of Sociology 51, no. 1: 107–123. 10.1080/000713100358453.

[shil70144-bib-0032] Lebovitz, S. , S. Lifshitz‐Assaf , and N. Levina . 2022. “To Engage or Not to Engage With AI for Critical Judgments: How Professionals Deal With Opacity When Using AI for Medical Diagnosis.” Organization Science 33, no. 1: 126–148. 10.1287/orsc.2021.1549.

[shil70144-bib-0033] Levy, I. , L. Bruckmayer , E. Klang , S. Ben‐Horin , and U. Kopylov . 2022. “Artificial Intelligence‐Aided Colonoscopy Does Not Increase Adenoma Detection Rate in Routine Clinical Practice.” American Journal of Gastroenterology 117, no. 11: 1871–1873. 10.14309/ajg.0000000000001970. 36001408

[shil70144-bib-0034] Litjens, G. , T. Kooi , B. E. Bejnordi , et al. 2017. “A Survey on Deep Learning in Medical Image Analysis.” Medical Image Analysis 42: 60–88. 10.1016/j.media.2017.07.005. 28778026

[shil70144-bib-0035] Mackintosh, N. , and N. Armstrong . 2020. “Understanding and Managing Uncertainty in Health Care: Revisiting and Advancing Sociological Contributions.” Supplement, Sociology of Health & Illness 42, no. s1: 1–20. 10.1111/1467-9566.13160. 32757281

[shil70144-bib-0036] Orlikowski, W. J. 1992. “The Duality of Technology: Rethinking the Concept of Technology in Organizations.” Organization Science 3, no. 3: 398–427. 10.1287/orsc.3.3.398.

[shil70144-bib-0037] Pesapane, F. , C. Volonté , M. Codari , and F. Sardanelli . 2018. “Artificial Intelligence as a Medical Device in Radiology: Ethical and Regulatory Issues in Europe and the United States.” Insights Into Imaging 9, no. 5: 745–753. 10.1007/s13244-018-0645-y. 30112675 PMC6206380

[shil70144-bib-0052] Petersen, A. 2018. Digital Health and Technological Promise: A Sociological Inquiry. Routledge.

[shil70144-bib-0038] Prasad, A. 2005. “Making Images/Making Bodies: Visibilizing and Disciplining Through Magnetic Resonance Imaging (MRI).” Science, Technology & Human Values 30, no. 2: 291–316. 10.1177/0162243904271758.

[shil70144-bib-0039] Reckwitz, A. 2002. “Toward a Theory of Social Practices: A Development in Culturalist Theorizing.” European Journal of Social Theory 5, no. 2: 243–263. 10.1177/13684310222225432.

[shil70144-bib-0040] Rose, G. , and D. P. Tolia‐Kelly . 2016. “Visuality/Materiality: Introducing a Manifesto for Practice.” In Visuality/Materiality. Images, Objects and Practices, edited by G. Rose and D. P. Tolia‐Kelly , 1–11. Routledge.

[shil70144-bib-0041] Sandfort, S. 2019. Bilder ohne Bildlichkeit? Zur Produktion und Rezeption radiologischer Bilder. Transcript.

[shil70144-bib-0042] Schatzki, T. R. , K. Knorr‐Cetina , and E. von Savigny , eds. 2001. The Practice Turn in Contemporary Theory. Routledge.

[shil70144-bib-0043] Taghiakbari, M. , Y. Mori , and D. von Renteln . 2021. “Artificial Intelligence‐Assisted Colonoscopy: A Review of Current State of Practice and Research.” World Journal of Gastroenterology 27, no. 47: 8103–8122. 10.3748/wjg.v27.i47.8103. 35068857 PMC8704267

[shil70144-bib-0044] Timmermans, S. , and A. Angell . 2001. “Evidence‐Based Medicine, Clinical Uncertainty, and Learning to Doctor.” Journal of Health and Social Behavior 42, no. 4: 342–359. 10.2307/3090183. 11831136

[shil70144-bib-0045] Timmermans, S. , and M. Berg . 2003. “The Practice of Medical Technology.” Sociology of Health & Illness 25, no. 3: 97–114. 10.1111/1467-9566.00342. 14498932

[shil70144-bib-0046] Ursin, F. , C. Timmermann , and F. Steger . 2022. “Explicability of Artificial Intelligence in Radiology: Is a Fifth Bioethical Principle Conceptually Necessary?” Bioethics 36, no. 2: 143–153. 10.1111/bioe.12918. 34251687

[shil70144-bib-0047] van Dijck, J. 2001. “Bodies Without Borders: The Endoscopic Gaze.” International Journal of Cultural Studies 4, no. 2: 219–237. 10.1177/136787790100400205.

[shil70144-bib-0048] van Dijck, J. 2005. The Transparent Body: A Cultural Analysis of Medical Imaging. University of Washington Press.

[shil70144-bib-0049] Wang, Y.‐P. , Y.‐C. Jheng , M.‐C. Hou , and C.‐L. Lu . 2024. “The Optimal Labelling Method for Artificial Intelligence‐Assisted Polyp Detection in Colonoscopy.” Journal of the Formosan Medical Association: (in press). 10.1016/j.jfma.2024.12.022. 39730273

[shil70144-bib-0050] Wittenberg, T. , and M. Raithel . 2020. “Artificial Intelligence‐Based Polyp Detection in Colonoscopy: Where Have We Been, Where Do We Stand, and Where Are We Headed?” Visceral Medicine 36, no. 6: 428–438. 10.1159/000512438. 33447598 PMC7768101

[shil70144-bib-0051] Witzel, A. , and H. Reiter . 2012. The Problem‐Centred Interview: Principles and Practice. SAGE.

